# A Tale of Enduring Myths: Buffon’s Theory of Animal Degeneration and the Regeneration of Domesticated Animals in Mid-19th Century Brazil

**DOI:** 10.1007/s10739-023-09742-8

**Published:** 2023-12-18

**Authors:** David Francisco de Moura Penteado

**Affiliations:** 1 University of São Paulo, São Paulo, SP Brazil; 2 King’s College London, London, United Kingdom

**Keywords:** Animal husbandry, Animal breeding, Agricultural modernization, History of science, Degeneracy, Climate determinism, Heredity, Animal biology

## Abstract

The long 19th century was a period of many developments and technical innovations in agriculture and animal biology, during which actors sought to incorporate new practices in light of new information. By the middle of the century, however, while heredity steadily became the dominant concept in animal husbandry, some policies related to livestock improvement in Brazil seemed to have been tailored following a climate-deterministic concept established in the mid-18th century by the French naturalist Georges-Louis Leclerc, the Comte de Buffon. His theory of animal degeneration posited, among other things, the necessity of recurrent crossbreeding to preserve animal species living in nonnative environments from climate-induced degeneration. Although largely discredited by the early 19th century, the teachings of the French naturalist seem to have found supporters in a Brazilian program to modernize national agriculture through the application of the natural sciences. Herein I examine the revival of Buffon’s theories in that government-sponsored program to improve animal husbandry and breeding techniques, including actual applications of this theory in the real world. Ultimately, I argue that Buffon’s theory of degeneration was used to tailor public policies and funding for the improvement of domesticated animals in Brazil between 1856 and 1860.

## Introduction

Hypotheses regarding the perverse influence of climate on the development, livelihood, and qualities of nonhuman animals and humans are as old as the history of Western thought (Sweet 2021; Glacken 1967). Throughout antiquity, the belief in the inexorability of the physiological influence of climate was widespread and shared by philosophers, historians, naturalists, and physicians (Gerbi 2010). The influence of the natural environment on animals, which encompassed climate, food availability and quality, topography, and soil properties, composed a particular part of those arguments, with warmer and arid regions causing sheep, for instance, to be “[…] born with wooly hair, crumpled horns, protruding lips, and wide nostrils; their extremities being as it were gnarled.”^1^

Some of these notions persisted and returned stronger in the early modern era (Heymann 2010). By the mid-18th century, a determinist view of climate as responsible for the distribution of species and shaping the qualities of living creatures gained strength in degeneration theory. This type of theory was developed by the French naturalist Georges-Louis Leclerc, Comte de Buffon (1707–1788), who claimed nonindigenous climates and diets had a negative impact on the development of animals and human beings, whose acquired characteristics were then inherited by later generations (Sloan 1974). Similar to philosophers from the Classical period, Buffon posited that animal species were inexorably subject to limited transformations in response to changes in climate. In some places, such as in the Americas, the natural environment was so averse to life forms that its native animal species were smaller, weaker, and fewer in number when compared to the animal species in Europe, Asia, and Africa. Alongside most of Buffon’s work, the degeneration theory became significantly popular among European animal breeders (Nelson 2021; Lush 1943) and intellectuals in the late 18th century, especially his views on the “New World” (Gerbi 2010, pp. 35–51).

While Buffon’s theory of animal degeneration and his claims about the Americas’ environment were mostly discredited by the early 19th century, with a growing trend among animal breeders placing more importance on inheritance (nature) than on the environment (nurture) (Pemberton et al. 2018; Olby 1996), some of these old Buffonian ideas seemed to have found supporters in Brazil. Launched in 1856 in the country’s capital, Rio de Janeiro, Buffon’s views were revived in the controversial book *Ensaio sobre a regeneração das raças cavalares do Império do Brasil* (Essay on the Regeneration of Horse Breeds in the Empire of Brazil), written by Brazilian engineer and natural scientist Frederico Leopoldo César Burlamaque (1803–1866). Heavily influenced by Buffon’s concept of degeneration, the author asserted that the poor state of domesticated Brazilian animals was caused by climate-produced degeneration, for which a solution could be found in a distinctive form of selective breeding.

Far from being an isolated endeavor, the publication of the book marked a broad movement of intellectuals and scholars to modernize Brazilian agriculture through the application of natural sciences.^2^ This reformist movement was a two-pronged undertaking, involving both an array of science communication efforts, and also the actual implementation of new production methods and techniques—which frequently required government support (Domingues 1995). While the latter was not commonly accomplished, the former became a fundamental part of this movement,^3^ often enacted by the translation and republication of European and American manuals and scientific literature. Standing out as a case that combined both aspects of this effort, the publication of Burlamaque’s book was followed by the creation of a government-sponsored program guided by Buffon’s theory to improve the condition—or regenerate—some of the Brazilian animal species.

The long 19th century was a period of many scientific developments and technical innovations in agriculture and animal biology, in which livestock improvement had become a complex and expensive enterprise, with the search for the most effective technique having great importance for economic actors.^4^ This essay aims to show the fragmentation and unevenness of the development and adoption of new scientific ideas internationally. While analyzing the circumstances associated with the application of Buffon’s degeneration theory in Brazil, I aim to contribute to the study of his views on animal biology, the role of the Brazilian government in supporting projects related to agriculture, and the history of Brazilian science.^5^ This essay analyzes these events in three sections. First, I examine the concept of animal degeneration through Buffon’s bibliography and in European and American publications on animal husbandry and agricultural manuals. In the second section, I investigate the resurgence of Buffon’s theories in Burlamaque’s book. Finally, in the third section, I explore measures taken to garner support for the proposal to regenerate domesticated animals, subsequent actions by the Brazilian imperial government to support it, and also contemporary criticisms of the scheme.

## Animal Degeneration in Buffon’s Natural History

Between 1749 and 1788, the French naturalist and director of the Paris Royal Botanical Garden Georges-Louis Leclerc, Comte de Buffon, published 36 volumes of his magnum opus *Histoire naturelle, générale et particulière, avec la description du Cabinet du Roi*, translated into English as *Natural History, General and Particular, with a Description of the King's Cabinet*.^6^ Buffon’s lifework, henceforth referred to as *Histoire naturelle*, was organized following the Enlightenment tradition of encyclopedic publications on natural history and covered a wide array of subjects, such as biology, geology, physics, and chemistry. Buffon’s work quickly grew in popularity and became one of the most widely read books of the 18th century (Huxley 2007; Roger 1997).

Among the novel ideas advanced by this work, Buffon was particularly proud of his theory of degeneration (Gerbi 2010, pp. 3–4). First presented in the fourth book of *Histoire naturelle*, whose theme was domesticated animals, Buffon argued that notwithstanding “[…] all creatures hav[ing] equally participated in the favors of creation,”^7^ they were prone to suffer significant modifications caused by the climate they inhabited (Buffon 1753, p. 383). Non-native environments, particularly diet and climate, had substantial effects on the development of animals and their offspring (Buffon 1753, pp. 215–223). As one example, Buffon held that generations of horses bred “in dry and light countries” were more “swift and vigorous” with “nervous legs and strong hoofs.” In contrast, generations of horses bred in “damp places, and in fat pasturage” developed undesirable characteristics such as “large, heavy heads, thick legs, soft hoofs, and flat feet” (Buffon 1753, p. 215). After a few generations, such deviant traits and attributes were accentuated due to the inheritance of acquired characteristics (Buffon 1753, pp. 221–223). Since the animal species’ characteristics conformed to their respective diets and climates, Buffon named this process “degeneration” (Buffon 1766, pp. 317–320), as a vitiated alteration from the original formation (Eddy 1994).

Having a natural environment that significantly affects animal development, offspring, and most importantly, negatively affects animal husbandry and stockbreeding, made these conclusions particularly significant for human affairs. Based on Buffon’s arguments, animal husbandry must consider the animals’ native diets and climate. However, whereas diet components could be more easily neutralized, it was not obvious what could be done to counter the influence of adverse climates on domesticated animals. Buffon argued, nonetheless, that this negative influence could be countered by human agency. Insofar as the influence of each climate on each animal species was not the same, because each one “gives a certain conformation of parts, which offends either by excess or defects” (Buffon 1753, pp. 217–218), it was possible to breed animal races from opposite climates to counterbalance their differences, thus neutralizing defects and improving quality. Thus, although degeneration was a natural process, there was a technique to replace degenerated traits with non-degenerated ones, a process called *renewal of the race* (Buffon 1753, pp. 217–218).

According to Buffon’s limited transformist theory (Caponi 2009; Galfione 2013), animal species had an original prototype from which their variations originated (Buffon 1753, pp. 215–217). The differences between animal groups of the same species were mainly caused by the progressive influence and subsequent degeneration in accordance with their environments, but these changes need not be permanent. “Spanish or Barbary horses,” said Buffon, “if the breed is not crossed frequently, become in France, French horses, in the second generation, and always in the third” (Buffon 1753, pp. 217–221). Therefore, in order to preserve the original traits of Spanish or Barbary horses in France, it was necessary to regularly have them breed with horses imported from Spain or the Barbary states, respectively. Moreover, in this situation, progeny quality could be improved if, rather than mating a newly brought stallion from Spain to a Spanish mare in France, a Spanish stallion was mated to a French mare, for the former would lack counterbalances from foreign climates (Buffon 1753, pp. 220–221). Thus, Buffon pointed to the need for crossbreeding alongside consideration of indigenous climates and those to which they were transported. And while he recognized the effects of human domestication of these animals on the degenerative process, it was not a sufficient condition for their degeneration (Buffon 1766, pp. 311–374).

In later books of *Histoire naturelle*,^8^ Buffon investigated the relationship between his theory and traveler reports and descriptions of animals from the Americas, as well as specimens in the King’s Cabinet of Natural History. Buffon’s findings were that not all areas of the world were created equally, and South America had poorer conditions for the development of living creatures, including for its native species (Dugatkin 2009). According to him, animal species in the American continents were generally “less active, less varied, and even less vigorous” (Buffon 1761, pp. 84–88). Having various animal species being transported from the “Old World" to the “New,” Buffon argued that most of them had become feebler, weaker, and smaller than their counterparts on the old continents. In his investigation, Buffon concluded that the cause of this degenerative process was the environmental conditions of the Americas, particularly temperature, moisture, topography, and soil quality. In his words, the cause for qualities of animated nature was inanimate nature (Buffon 1761, pp. 106–109). And although many animal species were successfully introduced to the Americas, such as hogs, sheep, and cattle, Buffon emphasized that they all appeared to have been negatively affected by the “New World’s” natural environment (Buffon 1761, pp. 70–72).

By the early 19th century, however, most aspects of degeneration theory related to animal husbandry were largely dismissed, particularly in respect to American fauna (Dugatkin 2009). Overall, a shift occurred towards placing the influence of heredity above that of the natural environment. This was particularly true for horse breeding, in which the worldwide success of the English Thoroughbred by the mid-18th century was founded on the belief that heredity was more important than the environment (Russell 1986; Weil 2020).^9^ The successful animal breeding practices developed by the British agriculturalist Robert Bakewell (1725–1795), with his breeding in-and-in technique; and the widespread introduction of the Spanish Merino sheep across Europe and North America—both in the second half of the 18th century—convinced animal breeders that stockbreeding could remain constant for as many generations as there was time to observe them (Wood 2007; Matz 2011). As observed by Robert Olby, “by the end of the 18th century breeders had found definite evidence that degeneration did not occur” (Olby 1996, p. 525).

European and American publications on animal husbandry and agriculture often dismissed Buffon’s theory and his recommendations in favor of climate-based crossbreeding of domesticated animals, instead embracing the notion that the environment played a lesser role (Wood 2007; Lehleiter 2014; Davidson 2009; Orel and Wood 1981). Some contemporary authors went as far as naming Buffon as responsible for spreading the misconception of climate-caused animal degeneration and the unrestrained usage of crossbreeding. In *Maison rustique du XIX siècle* (1835–1837), a French five-volume encyclopedia on agriculture authored by prestigious agronomists and members of agricultural societies in France, Buffon’s degeneration theory was promptly rejected,^10^ reflecting the predominant doctrine of racial constancy:

Buffon, and after him other naturalists, basing themselves on some isolated facts, will affirm that every race, multiplied by itself and kept pure of crosses, as well as every seed constantly cultivated in the same country, must necessarily degenerate. This theory, the natural consequence of which would be the complete degeneration of all wild animals, and of all plants, and which, moreover, is in obvious contradiction with the existence of the most perfect breeds of domestic animals, and the best varieties of cultivated plants, this theory has unfortunately been adopted by many of our breeders, and has often had sad results. (Bixio 1837, p. 376)^11^

Throughout the first half of the 19th century, numerous and significant technological changes in agricultural history occurred, in which agriculturalists experimented with new techniques and developed a better understanding of the role of heredity in animal breeding (Müller-Wille 2007; Jones 2016). By then, two breeding methods were established: (1) linebreeding, also known as purebred breeding and racial constancy, which relied on selective breeding of desired traits, and (2) the grading technique, following hereditary notions of blood fractions; -- with the latter remaining largely experimental until the end of the century (Derry 2015; Wood 2007). From Moravia (Wood and Orel 2005) to Brazil, authors reported the advantages of breeding practices that placed parentage and lineages over the natural environment.^12^ Moreover, animal husbandry practices that placed greater importance on good nutrition for animal development (Orel and Wood 1981), as well as the transplantation of animals through the concept of acclimatization, predicated on the premise that the influence of the environment could be overcome, were also being developed (Osborne 2001). Degeneration notions persisted, nevertheless. In the second half of the century, a new and broader concept of degeneration was developed. The term was adopted by other areas of knowledge and incorporated into medicine, law, and sociology, albeit with significantly different meanings and consequences (Sweet 2021; Cartron 2007; Chamberlain and Gilman 1985).

## The Tale of the Degeneration of Brazilian Horses

The noun degeneration and its counterpart in Brazilian Portuguese, *degeneração*, have their etymological source from the Latin word *degenere*, meaning “falling off from the generic or natural state” (Lawrence 2010). Throughout the 19th century, Portuguese dictionaries provided a very similar semantic sense, defining it as “the act of degenerating; change, which any body experiences in its intimate nature, or in its essence, which deteriorates; passage, or change, from the primitive state of a body to an inferior state, or worse” (Silva 1890, vol. 1, p. 596), or alternatively “less perfect, and seem to change into another species, or difference” (Silva 1823, p. 537). Thus, the term’s usage was relatively common and not restricted as a reference to Buffon’s theories of degeneration.^13^ Among the many ways it was used, there were several unrelated applications in agriculture: degeneration could refer to seed degeneration, animal degeneration due to inbreeding, crossbreeding, and poor nutrition. Therefore, for the sake of this article, and considering this semantic diversity and ambiguity, I will focus on the usage of “degeneration” with direct reference to Buffon.

While in the late 18th century Buffon’s degeneration was a reference for many Luso-Brazilian naturalists, such as Alexandre Rodrigues Ferreira (1756–1815) and João da Silva Feijó (1760–1824) (Ferreira 2022; Kury 2004), 19th- century Brazilian scholars and naturalists were not limited to his degeneration theory. On the contrary, the animal degeneration theory was absent from discussions related to animal husbandry, while some mainstream scholars openly rejected the theory that animals necessarily degenerated in the “New World.”^14^ Instead, Buffon was known for his contributions to the incipient field of biogeography and as a source of information on other topics of natural history.^15^ In Brazil the French naturalist nonetheless remained a prominent figure, whose lasting prestige may have been boosted by the strong French cultural influence in the country. And, rather than an outlier, the episode discussed here was one episode among many of the close Franco-Brazilian relationship and the strong French cultural influence over Brazil throughout the 19th century, which often included the exchange of technical-scientific knowledge (Hamburger et al. 1996; Perrone-Moisés 2013; Félix and Juall 2016). In fact, as will be seen later, both sides of the argument were either championed by French  people or by quoting French authors.

With this said, the resurgence of Buffon’s theory of animal degeneration in Brazil seemed to have been started by Frederico Leopoldo César Burlamaque, prompted by the civil association Sociedade Auxiliadora da Indústria Nacional (Society for the Promotion of the National Industry). According to the book preface and institutional information, on May 4, 1852, Miguel Calmon du Pin e Almeida, marquis of Abrantes (1796–1865), Brazilian senator and president of the association, requested that a project be formulated to improve horse breeds in Brazil. Burlamaque was thus chosen by the association’s board of directors to write a plan and instructions to solve a matter of utmost importance: the degeneration of Brazilian horses (Sociedade Auxiliadora... 1852, p. 431). The book was designed to be a manual on horse breeding and handling (Machado 1859), the first of many books commissioned by the association on agricultural subjects.

The Sociedade Auxiliadora da Indústria Nacional (hereafter Sociedade Auxiliadora) had been formally established in 1825 in Rio de Janeiro. Formed in the early years of Brazil’s independence by intellectuals and politicians but mainly composed of farmers, merchants, industrialists, and landowners, the association’s objective was announced by its title: the promotion of the national industry. Within the broad semantics of the word *indústria* (industry), the association aimed to promote economic growth through the application and dissemination of new technologies and techniques, particularly in agricultural production—then the engine of the Brazilian economy. The Sociedade Auxiliadora materialized its goals through several endeavors, most notably the publication of a periodical entitled *O Auxiliador da Indústria Nacional* (*The Helper of National Industry*), the publication and sale of scientific manuals on agricultural subjects, providing technical reports at the request of the Brazilian government, and the acquisition, distribution, and sale of domesticated animals. Among its members were some of the most prestigious Brazilian scientists and naturalists (Penteado 2022; Cribelli 2016, chap. 1).

One of these notable individuals was Frederico Leopoldo César Burlamaque.^16^ He was born in Lisbon, Portugal on November 16, 1803, and had been taken to colonial Brazil by his family at a young age, in 1806, due to his father’s appointment as Governor of the Captaincy (military governor) of São José do Piauí. Burlamaque followed the steps of his father in the military and joined the army at the age of fourteen, only retiring in 1855 with the rank of brigadier. It was also through the military that he obtained his formal education. Burlamaque studied at the Academia Militar da Corte (Military Academy) in Rio de Janeiro, where he majored in engineering, mathematics, and natural sciences, and subsequently obtained a doctoral degree in mathematical and natural sciences from the same institution. Later, in 1835, he was hired as an adjunct professor of exact sciences at the same institution, becoming an associate professor of mineralogy and geology in 1846 (Moreira 1866b; Silva 1870, pp. 403–405).

Sometimes named in the historiography amongst the most prestigious Brazilian men of science of the century (Lopes 1997), by the 1850s Burlamaque was an accomplished scholar with studies encompassing paleontology, chemistry, engineering, geology, and mineralogy (Fernandes et al. 2010; Marques and Filgueiras 2010). In June of 1847, he was nominated director of the National Museum, a position he held until his death on January 13, 1866 (Lacerda 1905, pp. 24–30). Burlamaque was also a member of several learned societies, being particularly active at the Sociedade Auxiliadora (Blake 1895, pp. 160–163). In 1849 he was elected secretary in perpetuity, becoming responsible for many of the association’s activities (Silva and Penteado 2017).

**Fig. 1:**
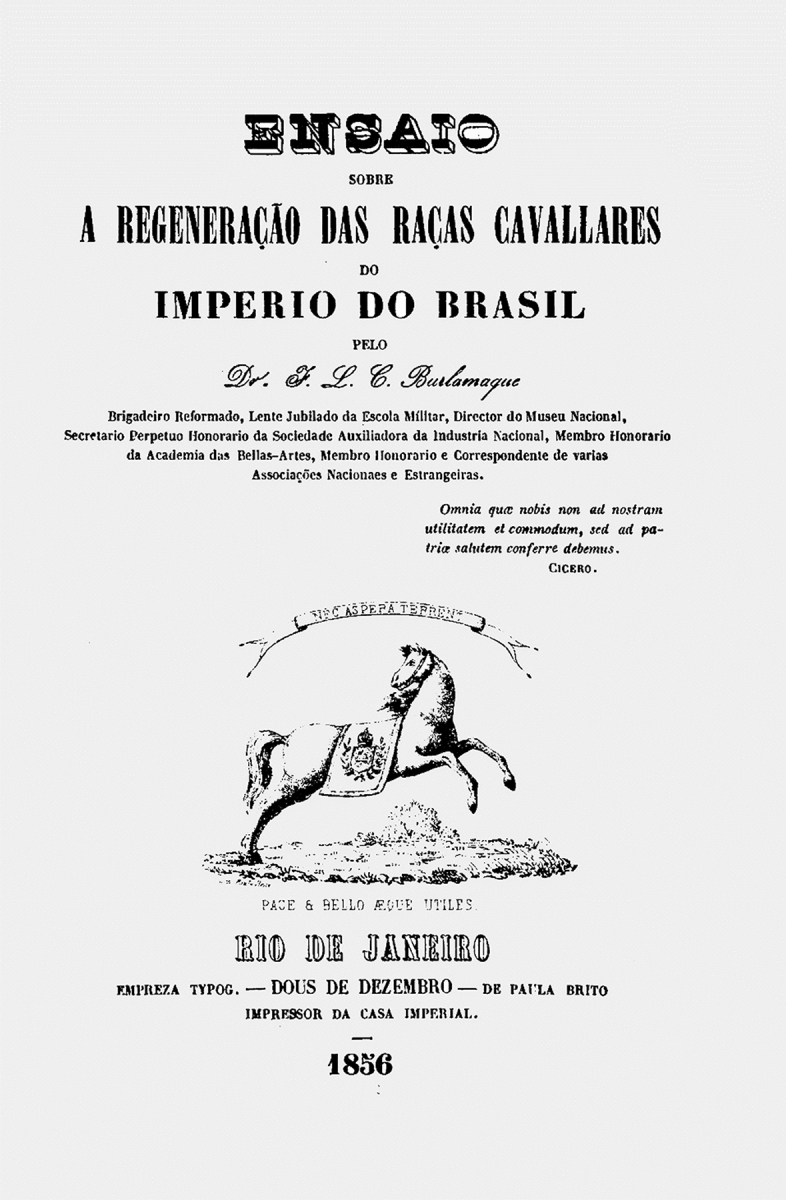
Title page of the book *Essay on the Regeneration of Horse Breeds in the Empire of Brazil*, by Frederico Leopoldo César Burlamaque (Burlamaque 1856a)

Then forty-seven years old in 1856, Burlamaque published the book *Ensaio sobre a regeneração das raças cavalares do Império do Brasil* (*Essay on the Regeneration of Horse Breeds in the Empire of Brazil*) (Fig. 1), a 133-page treatise on horse treatment, handling, and natural history, alongside a plan for horse improvement—or regeneration. Its objective, as requested by Sociedade Auxiliadora, was to present solutions to the question of the degeneration of Brazilian equine breeds. This problem was exemplified by a significant number of horses, from north to south of the empire, who were “ungainly and lacking in vigor,” lacking in stamina, and had a higher propensity to be affected by diseases (Burlamaque 1856a, pp. 6–7). According to Burlamaque, horse feebleness was an undeniable problem whose causes were twofold: first, lack of crossbreeding with more *perfect breeds* and, secondly, improper handling by horse keepers and owners. However, the first of the two causes seemed to be a sufficient condition, as he held that providing horses with only better living conditions would not be sufficient to avoid degeneration. In his words, all land-based species were doomed to degenerate if their *race mold* was not renewed (Burlamaque 1856a, pp. 1–2). The mention of perfect breeds seems to have been in reference to the Buffonian notion that the ideal form is dispersed across all regions of the earth; hence crossbreeding was required to restore breeds from degeneration (Buffon 1753, pp. 215–217).

Along the lines of Burlamaque’s proposal,^17^ the first part of the plan was for the Brazilian imperial government to mediate and provide transport for the acquisition of stallions of other breeds overseas to serve as breeding stock. After considering the attributes of a few famous horse breeds, the climates in which they were native, as well as their acclimation capacity, prices, and transportation costs, Burlamaque recommended purchasing the relatively small number of a hundred male horses from the Cape of Good Hope region, at the time the British Crown Colony of Cape. In his estimate, each horse could cost up to five hundred *réis* (500$000), with the total cost of 50:000$000 for a hundred horses; when including transportation costs, the final estimate was 80:000$000,^18^ or approximately £9,183 British pounds.^19^ In addition to individual animals, the government would have to acquire and send large quantities of plant seeds from the region to Brazil that could be used as fodder. To mitigate degeneration, their diet would have to be kept the same (Burlamaque 1856a, pp. 9–17).

Despite the hefty price, this would not be a permanent solution. Burlamaque reminded readers that this exercise would have to be repeated every few years, indefinitely, due to the inexorable nature of animal degeneration (Burlamaque 1856a, p. 12). This burden existed notwithstanding Brazil having significantly different climates within its territory due to its continental size, which could be employed to mitigate the degeneration effects. However, to some extent, since Brazil’s climate is characterized by a mostly tropical north to temperate zones in the south, the process of periodically sending northern horses to the south and southern horses to the north might also be useful to mitigate the degeneration caused by the climate (Burlamaque 1856a, p. 101). Burlamaque also briefly considered other proposals to improve equine living conditions but dismissed most of them. Regarding the creation of horse stud farms by the Brazilian government, he contended that it had prohibitive costs and that it was being phased out in other countries (Burlamaque 1856a, p. 7). Instead, Burlamaque (1856, p. 17) recommended that provincial governments establish a program of prizes for successful horse breeders following the premises outlined in the book.

Therefore, the central aspect of the plan was to acquire and transport individual horses and have them breed with horses in Brazil. The missing part of the scheme, however, left unexplained in the first part of the essay, is the nature of animal degeneration and the proposed solution. Only later in the essay, in its second part, Burlamaque explained the causes of the so-called animal degeneration. Following the notion of inheritance of acquired characteristics and Buffon’s teachings on the natural history of horses (Buffon 1753, pp. 174–376), Burlamaque argued that climate and diet were the main factors responsible for the degeneration of horses’ physical attributes, then proceeded to translate and quote Buffon, word by word, on the degeneration of domesticated animals (Burlamaque 1856a, 99–101; Buffon 1753, pp. 215–217). First, he laid out the theoretical foundation for Buffon’s degeneration theory:

There is in nature a general prototype of each species, says the illustrious Buffon, from which each individual is modeled, but which seems in procreation to be debased, or improved, according to its circumstances, insomuch, that in relation to certain qualities, there is a strange variety in the appearance of individuals, and at the same time a constant resemblance in the whole species. [...] and what is singular, the model of what is handsome and excellent is dispersed through all parts of the earth, and that in each climate there is a portion thereof, which perpetually degenerates, unless united with another portion taken from a distant country [...] To have fine horses, dogs, etc., it is proper for the males and females to be of different countries. Without this being attended to, corn, flowers, and animals, will degenerate, or rather take so strong a tincture of the climate as to deform and bastardize the species; the form remains, but disfigured in all the lines which are not essential thereto; by mixing, on the contrary, the kinds, and above all, by crossing their breed with foreign species, their forms seem to become more perfect. Burlamaque 1856a, pp. 99–100, translation mine.

Overall, the procedure outlined was very similar to the example given by Buffon in the fourth volume of the *Histoire naturelle* with the Spanish and French horses. Deeming climate-caused degeneration to be a natural process, Burlamaque reaffirmed the necessity of crossbreeding. In his argument, and paraphrasing Buffon’s words, conserving the breeds contributed to animal degeneration, whereas crossbreeding them countered the degenerative effects caused by the climate. Likewise, climate characteristics were also significant when choosing which breeds to cross. Following Buffon’s approach, Burlamaque concluded that the correct answer lay in balancing the effects of opposing climates.

Consequently, in order for the horse breeds not to degenerate completely, it is necessary to cross the breeds, instead of conserving them; and this crossing must be renewed in every generation; causing the mares of the country to be covered by foreign horses, and afterwards sending for foreign mares to have them covered by horses of the country. It is also not indifferent to send for mares or horses from anywhere, but to bring these animals from countries whose climates are opposite to the country where they are trying to improve the breeds. Each climate, through its influences and those of nutrition, gives a certain conformation that sins by excess, lack, or some deed: in a hot climate there will be in excess what will be lacking in a cold country, and vice versa, in a way that must there is compensation when two animals from opposite climates come together. (Burlamaque 1856a, pp. 100–101, translation mine).

For the sake of clarity and as a comparison tool to Buffon’s arguments, Burlamaque’s arguments can be summarized in the following premises and conclusion:

- Degeneration occurs if animals are not regularly crossed with different and non-degenerate breeds.
- Climate is the single most important cause of the degenerative process.
- Their offspring suffer greater degenerative effects, with each generation suffering more than the last due to the inheritance of acquired characteristics and longer exposure to the climate.
- Over time, their original attributes are lost.
- By regularly importing non-degenerate individual animals from opposite climates and having them crossbreed with degenerate specimens, the latter traits will be replaced by the former.
- Recurrent crossbreeding with specimens from opposite climates counters degeneration.

Conversely, though degeneration was extensively defined and described in Burlamaque’s proposal, regeneration was only defined as opposed to the former. Regeneration, or *regeneração* in Brazilian Portuguese, had primarily numerous religious meanings, commonly in reference to the idea of being born again after receiving baptism. But outside this scope, it was broadly defined as an effort towards amending and correcting something (Silva 1890, vol. 2, p. 682). While the term did not seem to make direct reference to a Buffonian concept related to animal breeding, it appeared to have referred to the concept of restoring to animals the traits of their original prototype formation. In other words, it implied the ability to reverse some of all the modifications caused by degeneration (Nelson 2021).

Furthermore, despite his explanation of the degenerative process, the state of affairs criticized by Burlamaque is obscure. The available documentation does not allow us to build a comprehensive picture of the animal breeding practices in Brazil. Instead, contemporary authors criticized the poor quality of Brazilian horses and the lack of coherence and consistency breeding methods (Adet 1858; Abreu e Souza 1875).

## Government-Sponsored Projects for Animal Regeneration

In 19th-century Western society, the utilization of horses was ubiquitous, and this was no different in Brazil: horses were a fundamental component of the Brazilian economy and society. They were used to transport people and goods, enabling relatively fast long-distance logistics, communication, mail, hunting, leisure, sports, military operations, and employed in many agriculture-related roles (Thompson 1976; Soppelsa 2011; Guest and Mattfeld 2019). Brazil was also a latecomer to the construction of railroads, with a low level of penetration and seriously uneven distribution of railroad tracks (Leff 1972; Absell and Tena-Junguito 2017), thereby increasing its relative dependency on pack animals. Furthermore, as Burlamaque stated, even the Brazilian military struggled to find horses suitable for its needs. The importance of horses for the military, from their use in cavalry to logistics, turned the problem into a matter of national security and sovereignty. Given these crucial roles, Burlamaque argued that the Brazilian government had the duty to intervene and pursue a solution (Burlamaque 1856a, pp. 6–8). At the time, calls for government intervention in matters related to horse breeding, and actual interventions, were not out of the ordinary in Europe and the Americas. There was a widespread concern from governments with the number and quality of horses, particularly warhorses (Guest and Mattfeld 2019; Roche 2008).

The first indications of government support for the “horse regeneration” proposal appeared as soon as the book *Essay on the Regeneration of Horse Breeds in the Empire of Brazil* was published, in early 1856, with printing expenses paid for by the Brazilian government (Silva 1870, p. 404; Blake 1895, p. 162; Brazil 1857, p. 118). Although the details of the negotiations are unknown, the nature of Sociedade Auxiliadora’s relationship with the government is well understood. As briefly described earlier, the association enjoyed the rewards of its close ties with the Brazilian imperial government and several provincial governments, as a beneficiary of public funds, and also as a regular advisory body on technical-scientific subjects. Through this special relationship with government officials, many of whom were members of the association, it sometimes managed to conquer enough influence to shape public policies (Penteado 2022).

While previously government support for agricultural improvement projects had been limited, no time was wasted in implementing this proposal (Penteado 2022). Later in 1856, the Sociedade Auxiliadora published a second edition of Burlamaque’s book (Burlamaque 1856b), as well as printed its main content in its monthly periodical.^20^ In the new edition, a significant amendment was made, which recommended the importation of horses from Hanover, Mecklenburg, and Holstein, apparently for financial reasons (Burlamaque 1856b, p. 3; Brazil. Ministério do Império 1858, p. 61). Then, in August 1857, the association sent a copy of the book and a letter to all provincial governments, both the executive and legislative branches, outlining their plan for the regeneration of horse breeds, whose very existence was supposedly under threat of extinction. The letter claimed that although “almost all our animal races are more or less degenerated,” the role of horses in Brazilian society and economy made it a pressing issue (Abrantes et al. 1857, pp. 428–429). The objective, therefore, was to gather support from the provincial governments to implement Burlamaque’s proposal.

Both editions of Burlamaque’s book seemed to have been received positively. From the north to the south of the empire, provincial governments received copies of the essay, and some even rejoiced that such a matter was receiving attention. For instance, in the province of Ceará, the local government attempted to import several different breeds of domesticated animals to improve local animal husbandry, among which were horses (Ceará 1858, pp. 26–27; Maranhão 1858, p. 14; Rio Grande do Sul 1860, pp. 59–60). Due to the book’s nationwide distribution, it quickly made its way to local newspapers and its content was republished a couple of times.^21^

In September 1857, only one year after the publication of the proposal, the national budget law for the 1858–1859 fiscal year brought an authorization for the government to spend up to forty *contos de réis* (40:000$000) on the improvement of the horse breeds and the acclimatization of dromedaries, or £4,431.^22^ The proposal for equine regeneration had been introduced to the Chamber of Deputies by the Sociedade Auxiliadora in June of that year, and it was approved as soon as August (Brazil. Câmara dos Deputados. 1857, vol. 3, pp. 67, 274). The funds were allocated to the Secretary of State for Imperial Affairs (Secretaria de Estado dos Negócios do Império), the same ministry that had paid for the publication of the first edition of Burlamaque’s book. Budgetary information for the 1857–1858 fiscal year brought the information that 17:777$778 were spent on the purchase and transportation of the horses (Brazil 1860, p. 116) or approximately £1,969. The funds were used to pay for twelve stallions from the region of Germany (Brazil 1857, pp. 61–62; Fausto 1859, p. 32); nine of which made it alive to Brazil, but two others died soon after arriving in late 1858 (Brazil. Ministério da Agricultura 1862, pp. 5, 10–11; Brazil 1859, p. 61). The seven remaining stallions were sold in the southern provinces of Minas Gerais, São Paulo, Paraná, and Rio Grande do Sul (Brazil. Ministério do Império 1859b, p. 35).

In 1858, it was announced that the provinces of Minas Gerais, Ceará, and Rio Grande do Sul had accepted Burlamaque’s proposal and decided to contribute to the regeneration program. In total, Ceará contributed 20:000$000; the Rio Grande do Sul with 16:000$000; Minas Gerais with 12:000$000, with an additional 4:000$000 from unspecified provinces. Together, provinces contributed 52:000$000 for the project (Machado 1859, pp. 166–167), or £5526 at the time. The actual destination of this money is unknown, but it indicates that the problem described as animal degeneration was recognized by part of the provincial political class. A year later, in 1859, the government issued an executive order allowing an extra credit of 23:193$000 to the 1857 law.^23^ The budgetary information for 1858–1859 elaborated that 12:787$839, or £1358, were spent on the equine regeneration program (Brazil. Ministério do Império 1861, p. 110), which was employed to purchase “African horses” (Arabians) from Algeria, the same place of origin of the aforementioned dromedaries. The second batch of horses disembarked in Rio de Janeiro in the second half of 1859 (Vogeli 1862, pp. 18–26), supposedly intended for the northern provinces (Brazil. Ministério do Império 1858, p. 61). With this additional purchase, the central government's total outlay for the program came to 30:565$617 (see Table 1), or £3327.^24^

**Table 1:** Expenditure related to animal regeneration by the imperial government, 1857–1859

1857–1858^a^	
Purchase of twelve (12) horses, transportation costs, and upkeep.	17:777$778
1858–1859^b^	
Purchase of thirteen (13) horses	8:888$888
Transportation and upkeep	2:848$951
Envoy’s payment	1:050$000
Total (*milréis*)	30:565$617

^a^Brazil. 1860 *Balanço da receita e despeza do Imperio no exercício de 1857–1858*. Rio de Janeiro: Typograhia Nacional, p. 116

^b^Brazil. 1861. *Balanço da receita e despeza do Imperio no exercício de 1858–1859*. Rio de Janeiro: Typograhia Nacional, p. 110

**Fig. 2:**
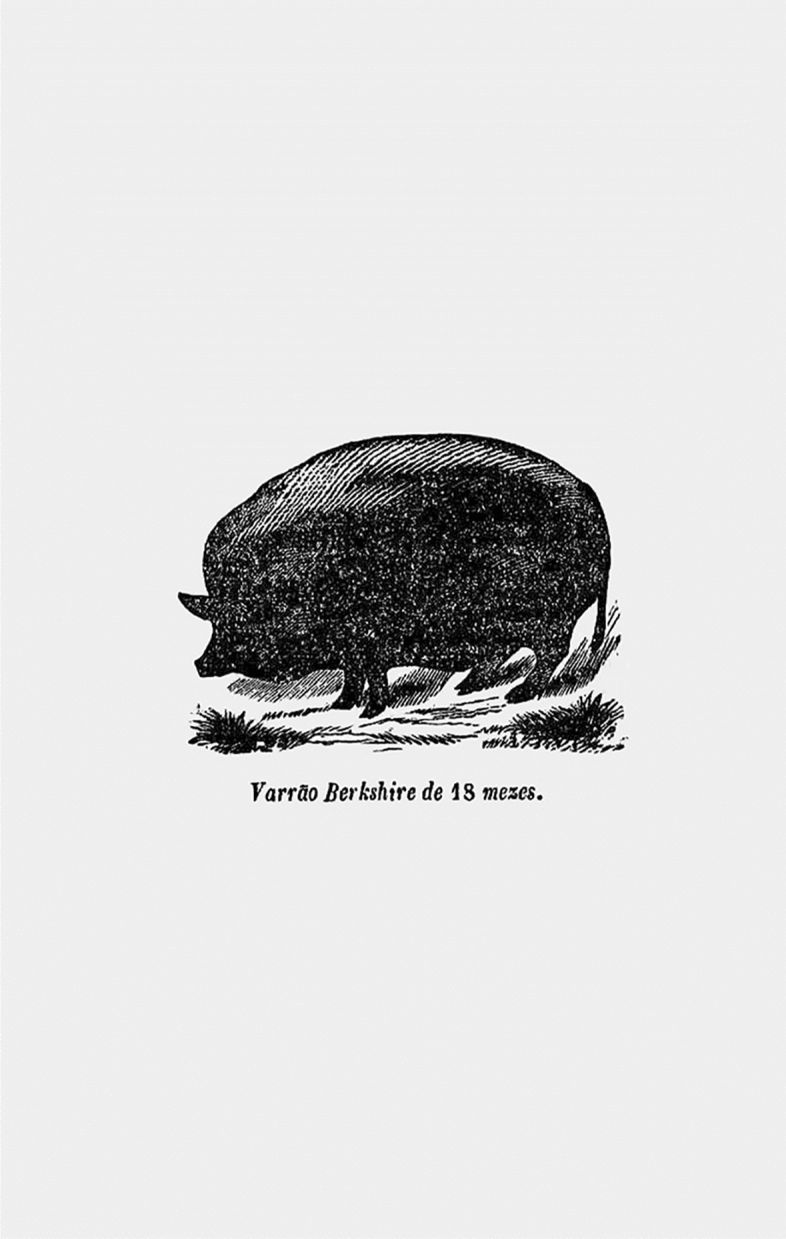
Illustration of an 18-month-old male Berkshire pig from Azevedo’s book (Azevedo 1860, p. 8)

While the horse importation scheme appears to be the last to receive direct government funding, it was not the last to receive support from government officials. Also in 1859, through the Brazilian diplomatic mission in London, it was arranged to purchase 40 pigs (six males and 34 females) of the English Berkshire breed (Fig. 2), which were to be shipped and sold in Minas Gerais by the Sociedade Auxiliadora. The proposal originated from the association’s recently created Commission for the Improvement of Animal Breeds.^25^ According to the Commission, the degeneration of Brazilian swine breeds was a growing problem, with the arguments revolving around the importance of pigs to commerce and the diet of Brazilians. In May 1860, however, when the ship arrived at the port of Rio de Janeiro, out of six male pigs and 34 female pigs shipped from the United Kingdom, only one male and 23 females made it alive. The association spent 3:136$647 (£327) on their purchase and transport, with the sale of the remaining pigs generating revenue of only 2:378$530 (£255) (Azevedo 1860, pp. 33–36).

**Fig. 3:**
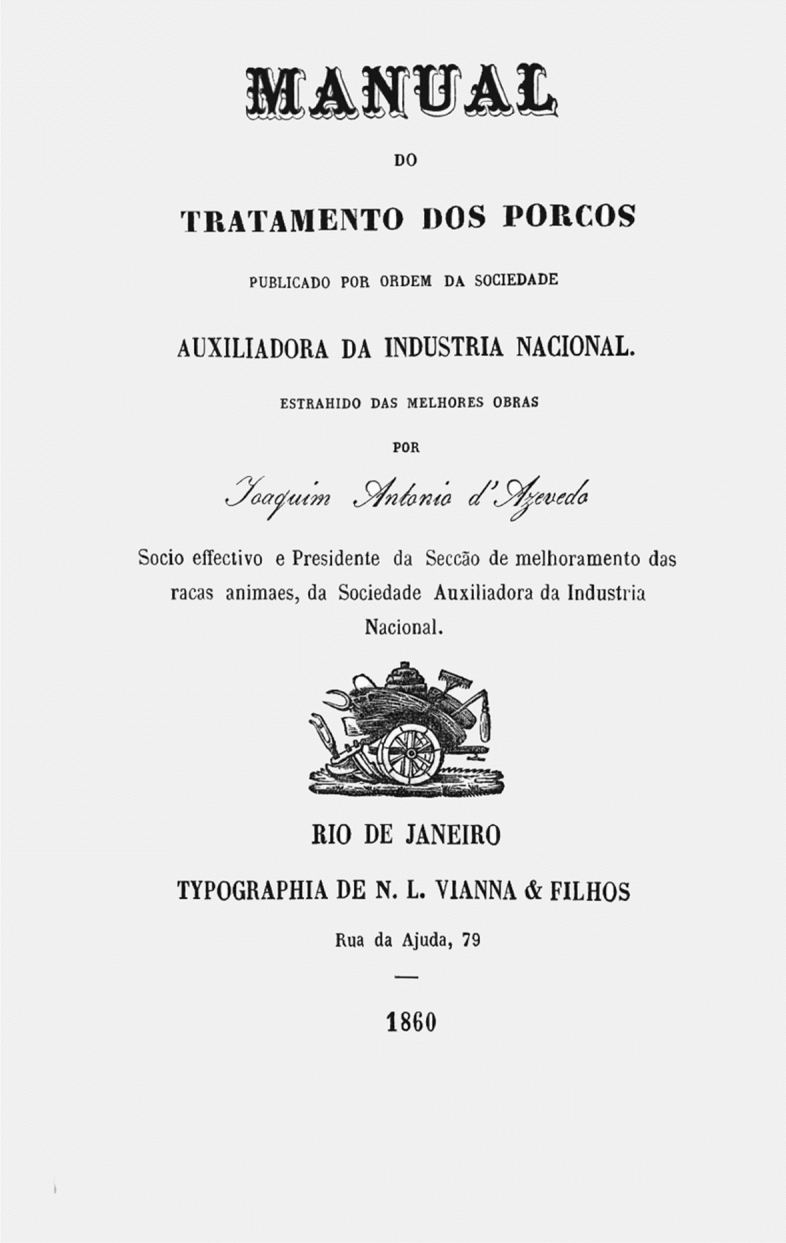
Title page of the book *Manual for the Care of Pigs*, by Joaquim Antônio de Azevedo (Azevedo 1860)

Together with purchasing English pigs, the Commission also proposed making a manual teaching the principles related to pig farming—along the same lines as Burlamaque’s essay. The responsibility to compose the manual fell to Joaquim Antônio de Azevedo (1819–1878), then president of the Commission and a Treasury functionary. One year later, in 1860, he published the *Manual do tratamento dos porcos* (*Manual for the Care of Pigs*), a 40 page brochure with information extracted from other works on the subject of pig farming, proper handling techniques, and those related to specific characteristics of the Berkshire breed, such as feeding, breeding, ideal sizes, etc. (Fig. 3). The main argument of the manual, however, and the one used to support the importation of pigs was Buffon’s animal degeneration theory. Azevedo claimed that the degeneration of the Brazilian swine breeds was well-known and widespread throughout Brazil. According to him, in some provinces, such as in Minas Gerais, “the best swine breeds have almost completely disappeared.” And although he seriously considered the role of undernutrition and epizootic diseases, he turned to Buffon’s animal degeneration for a final answer (Azevedo 1860, p. 1).

Relying on the theoretical groundwork laid by Burlamaque in his book, Azevedo argued that degeneration had two causes: lack of crossbreeding with other and more perfect races; and due to insufficient consideration for inbreeding, breeding age, and the pigs’ qualities by their owners and caretakers. While quoting Burlamaque, Azevedo stated that “all races of land animals degenerate, and wither from generation to generation, if the internal mold of the races is not renewed from time to time” (Azevedo 1860, pp. 1–3). Thus, following the lines of Burlamaque’s essay, Azevedo’s proposal aimed at the importation of more perfect swine breeds to avoid their complete degeneration.

The importation of the Berkshire pigs was the last known project related to Buffon’s animal degeneration supported by the Brazilian government, although no official statement was made regarding its interruption. The Sociedade Auxiliadora continued to serve the role of an advisory body to the government (Penteado 2022) as well as publishing manuals on agricultural topics, many of which were authored by Burlamaque.^26^ Notwithstanding, in the years following the publication of Burlamaque’s book, articles related to animal husbandry in the periodical *O Auxiliador da Indústria Nacional* seemed to have ignored the climate determinism behind the animal regeneration program and the inherent advantages of crossbreeding, previously championed by the association. Instead, they argued for the benefits of racial constancy and the importance of correct animal husbandry practices to the development and improvement of domesticated animals. Furthermore, Burlamaque's successor as editor of the association’s periodical directly criticized horse and pig regeneration schemes (Moreira 1866a; Sociedade Auxiliadora 1870). Whether for distinct priorities,^27^ reaction to criticism, or other reasons, the Buffonian premises of the animal regeneration program lost momentum a few years later. In 1869, the Commission for the Improvement of Animal Breeds was dissolved (Sociedade Auxiliadora 1869).

Indeed, in the years following the animal regeneration scheme there were at least two significant rebuttal attempts against it, with both arguing that the degeneration problem was not a question of climate or breeding. In 1858, Carlos Emílio Adet (1818–1867)^28^ published a 263-page book entitled *Zootecnia aplicada. Hipologia* (Applied Zootechnics. Hippology). Adet’s goal was to publish a scientific manual for horse breeders and, at the same time, also as a rebuttal to Burlamaque’s proposal and its theoretical basis.^29^ Adet deemed Burlamaque’s proposal a “radically erroneous system, the son of the hypothesis most contrary to scientific data and today disproved by experience” (Adet 1858, pp. 3–4), and blamed Buffon for such antiquated misconception.^30^ According to Adet, the quality of horses in Brazil was determined by its overall economic conditions. Insofar as he seemed to agree with the notion of hereditary transformation in response to the environment, his conclusions were not the same because if an English stallion were to mate to a Brazilian mare, there was no reason to suppose the traits of the former would prevail over the latter in their progeny (Adet 1858, pp. 4–5). Likewise, he argued that the quality of soil and roads, the treatment and nutrition of animals, together with animal handling traditions were more important than the influence of the climate (Adet 1858, pp. 25, 61–63, 79–84). For that reason, he held that the focus should have been on enhancing zootechnical practices, establishing artificial meadows, horse stud farms, and adopting improved plowing instruments to improve animal nutrition (Adet 1858, p. 101).

A more convincing critique appears to have come from a foreign veterinarian who had recently arrived in Brazil. After the second importation had taken place, French veterinarian Félix Vogeli (1832–1910) defended a different approach to the equine degeneration problem.^31^ At the time, Vogeli was an equitation and hippiatric professor at the Escola Central (Central School) in Rio de Janeiro.^32^ Intriguingly, he was the envoy who transported to Brazil the aforementioned dromedaries and the second batch of horses (Vogeli 1862, pp. 18–26). Previously, in 1856, he was hired by the Secretary of State for War Affairs (Secretaria de Estado dos Negócios da Guerra) to assess the condition of domesticated animal species, particularly horses (Brazil. Ministério da Guerra 1857). Vogeli’s proposal was initially submitted to a ministerial cabinet that forwarded it in 1861 to the Sociedade Auxiliadora’s Commission for the Improvement of Animal Breeds (Brazil. Ministério do Império 1861). In his unapproved proposal, reviewed in 1863, Vogeli advocated creating a veterinary school, a horse stud farm, and improved fodder production for animal nutrition. Regarding the regeneration policy, he called it a waste of time and money (Azevedo and Conceição 1863). Four years later, in a surprising turn of events, Vogeli became the president of the same commission (Sociedade Auxiliadora 1867, p. 93).

It remains to be seen whether this criticism had any significant influence on subsequent public policies regarding animal husbandry. Nevertheless, in hindsight, the following decades after the end of the program saw the beginning of a plethora of other initiatives related to animal husbandry by the imperial government. For instance, in 1873, after the War of the Triple Alliance (1864–1870), the Secretary of State for War Affairs decided to create a horse stud farm in Rio Grande Sul,^33^ which would apply the best-known zootechnic system, and production of fodder to improve animal nutrition (Brazil. Ministério da Guerra 1874, pp. 68–69). The stud farm was inaugurated in the early 1880s, with others to follow (Brazil. Ministério da Guerra 1886, pp. 54–56). Investments in programs related to the improvement of other domesticated animal species also grew steadily. The then-newly created Secretary of State for Agricultural, Commerce, and Public Works Affairs (Secretaria de Estado dos Negócios da Agricultura, Comércio e Obras Públicas), established in 1860, sponsored several projects for the creation of animal studs, model farms, the introduction of new breeds from neighboring countries, and zootechnical education. Amongst its objectives was the establishment of veterinary schools, which saw fruition by the end of the century.^34^

## Conclusion

While this article has investigated the Buffonian influence on the Brazilian scientific thought and public policies related to animal husbandry, broader implications encompass Brazilian science, the longevity of Buffon’s *Histoire naturelle*, the resilience of climate determinist notions, the circulation of scientific knowledge between Brazil and France, and the obsolete nature of some public policies related to agricultural modernization. The Brazilian animal regeneration program was at the crossroads between the development of modern animal breeding techniques, natural history, and the global exchange of knowledge. Although the importation of domesticated animals was widespread in many countries and an important component of selective breeding methods by the mid 19th century, climate no longer played an inexorable role in tailoring this practice. Buffon’s approach, as defined by Burlamaque, which consisted of the need for (a) recurrent breeding, (b) with other breeds, and (c) oriented by ancestral climate, was generally perceived as outdated. At that time, heredity was the guiding force for most animal breeders conducting their businesses and governments in related policy making (Cobb 2006).

Regarding the Buffonian influence, the verbiage and arguments used by Burlamaque in the *Essay on the Regeneration of Horse Breeds in the Empire of Brazil*, alongside his references, direct quotes from Buffon’s *Histoire naturelle*, and mentioning its fourth volume—*Les animaux domestique*—in the book bibliography, leave little doubt of his endorsement of the theory of animal degeneration, at least as far as the fourth volume went. By relying on Burlamaque’s theoretical foundation, Azevedo’s *Manual for the Care of Pigs* seemed to have followed the same path: firstly, by interpreting the animals’ poor conditions as an unavoidable degenerative problem caused by lack of crossbreeding, and then by adopting the notion that climate-guided crossbreeding was the only solution to improve their condition. It is less clear, however, how far Burlamaque’s argument went and how much he agreed with Buffon’s views on animal degeneration, whether it included “New World” biogeographical degeneration or not. Burlamaque never made any direct reference to alleged deficient environmental conditions in Brazil.

The question of why Burlamaque relied on Buffon’s *Histoire naturelle* to address local problems with animal husbandry also remains unanswered. Given the events and circumstances described, his proposal seems to be an outlier within well-established viewpoints. Brazil was not disconnected from the circulation of information about the latest developments in animal breeding practices, which are highlighted by other publications about animal husbandry and by the scheme to acclimatize dromedaries. Although there does not seem to have been a retraction of the concepts expressed in the animal regeneration program, their influence was short-lived. While the central part of the plan submitted to the government was perceived as unsound, the same cannot be said about the rest of Burlamaque’s essay or Azevedo’s manual. There were many important and useful lessons about equine and swine domestication, respectively, including anatomy, feeding and nutrition, handling, preventive medicine, healthcare, castrations, etc. These lessons were fundamental for animal breeders with little to no access to specialists to assist them (Germiniani 1998).

On the other hand, information regarding the poor condition of domesticated animals seemed reliable, despite the shortage of research on the subject.^35^ Poor animal husbandry practices, nevertheless, as assessed by Carlos Émile Adet and Félix Vogeli, are a far more likely explanation. In a country known for its archaic agricultural production methods, overconcentration of land ownership, and widespread monoculture, where modern techniques had not been widely adopted, it is not unlikely that the claims about the “degeneracy” of domestic animals were valid.^36^ To the same extent that many Brazilian farmers frequently failed to employ fertilizers, a waged and trained workforce, agricultural tools and instruments, systematic drainage, crop rotation, remedies and techniques to combat plant pathologies, and basic principles of chemistry, botany, and meteorology; they likely failed to employ modern husbandry techniques, such as selective breeding, proper nutrition, and zootechnical practices to increase animal productivity and life expectancy (Dean 1971; Leff 1972; Eisenberg 1974; Stein 1985; Lourenço 2001; Cribelli 2016, chap. 4). Whether due to a lack of information, financial restrictions, or a mere preference for tradition over technological innovations, the results seem to indicate that they were not being utilized. Therefore, it is not surprising that in their pursuit of technological modernization, the individuals involved in science popularization could have committed mistakes, especially considering the low volume of local scientific production and, in corollary, their reliance on European and American scientific production (Gutiérrez-Maya et al. 2021).

One of the conclusions of this research is that outdated scientific information seems to have been part of a program to improve domesticated Brazilian animals, which seems to be an insufficiently explored subject. Whereas scholars have often examined and, not rarely, even praised the efforts of individuals involved in science communication and popularization in 19th-century Brazil and Latin America (Pallares-Burke 1998; Britto 2002; Vergara 2008), not as much research has been done about the scientificity and contemporaneity of the claims made in their publications by scientific standards of their time. In the case of the regeneration of domesticated Brazilian animal species, information was not only used in science communication, but also the creation of government-sponsored schemes. Finally, this study bears on the larger issue of relationships between science and government, and how they are shaped by local views on biology.

## Footnotes

1 Statement attributed to Posidonius by Strabo, see Strabo, *Geography*, 2.2.3 (Strabo and Jones 1924, pp. 365–371). Also available in the Perseus Digital Library.

2 The term modernization is used in reference to the broad movement of Latin American groups who sought to adopt European production methods, technologies, and political ideas in their countries (Moya 2018). Throughout the 19th century, Brazilian authors involved with science popularization repeatedly described individuals in agriculture as hostages to routine and habit, whose archaic techniques held back the country's development. This position is closely related to the perception that science popularization in the 19th century was characterized by mechanisms of demarcation and discrimination between science and traditional knowledge (Silva 2020; Bensaude-Vincent 2009).

3 Science communication and popularization efforts were part of a significant number of Brazilian publications in the early 19th century, see Massarani and Moreira (2016).

4 See, for example, the Uruguayan case, Travieso (2022).

5 I borrow Topham’s (2009, pp. 1–20) argument that science popularization should be understood as an integral part and one of the most relevant paradigms when investigating the history of science.

6 The complete version of *Histoire naturelle* has forty-four volumes, however. After Buffon died in 1788, his colleagues continued his work and published eight more volumes.

7 English translations of Buffon are mostly based on James Smith Barr’s translations of *Histoire naturelle* (Buffon 1792).

8 Animal degeneration was addressed in different volumes of the *Histoire naturelle*, but mainly covered in three of them: in the fourth volume, in the chapters *Les animaux domestique. Le cheval* (Of domesticated animals. Horse), and in *L’Asne* (The Ass); in the ninth volume, *Animaux de l’ancien continent* (Animals of the Old Continent), *Animaux du nouveau-monde* (Animals of the New World), and *Animaux communs aux deux continents* (Animals common to both continents); and in the fourteenth volume, *De la dégénération des animaux* (Of the degeneration of animals).

9 Recently, Weil (2020) addressed this transition in French thought on horse breeding. from the Buffonian crossbreeding approach to the adoption of the theory of racial constancy.

10 Excerpts of *Maison rustique du XIX siècle* were translated and published in the periodical *O Auxiliador da Indústria Nacional* in 1838 (Moll 1838).

11 Regarding horse breeding, their recommendation was the opposite of Buffon's: “The more constancy a race has, the slower the degeneration” (Bixio 1837, p. 374–5).

12 For a sample of related articles, see Rio de Janeiro 13 de Fevereiro de 1828, 1828; Anon. 1812; Siqueira 1812; Anon. 1835; Anon. 1836; Taunay 1839, pp. 99–101.

13 As remarked by Borges (1993, p. 236), “in its late-19th-century heyday, degeneration was an inclusive, much-abused, catchall term.”

14 When mentioned, it was often openly rejected. For instance, in *O Fazendeiro do Brazil* (1806, p. 52), José Mariano da Conceição Veloso (1742–1811) asserted Brazil did not have an unsuitable climate for horses or other domesticated animal species.

15 For examples of mid-19th Brazilian scholars debating Buffon’s New World degeneracy theory, see Figueiredo et al. (2017); Cerqueira e Silva (1849); also see note 13.

16 Burlamaque’s middle name and surname had different spellings in contemporary sources. César was sometimes spelled as Cezar, and Burlamaque as Burlamaqui.

17 Burlamaque’s book was divided into two parts, each with two chapters. The first part described the problem and the problem of equine breed degeneration, whereas the second part covered general information on their natural history. In the first part, which is the focus of this article, Burlamaque sought to answer three questions: (1) What are the best equine breeds, and what should be their country of origin? (2) What is the best way to acquire and distribute them between the provinces of the Empire? (3) How much would it cost to buy, import, and upkeep the horses and how would it compare to the benefits?

18 The Brazilian currency was the real, or *milréis*, the effective unit of currency, expressed as 1$000. In values equal to or greater than one thousand, the *cifrão* (akin to the dollar sign) is inserted after the thousands digit; for values equal to or greater than one million, a colon is inserted after the millions digit. One *conto* equals a thousand *milréis*.

19 All exchange rates are obtained from *Estatísticas Históricas do Brasil* (Brazil 1990, pp. 568–569).

20 The essay was published in twelve issues between October 1856 and September 1857 of the periodical *O Auxiliador da Indústria Nacional*. The periodical was also distributed nationally by the central government.

21 Positive reviews about the book could be found in newspapers and government reports in the provinces of Paraná, Minas Gerais, Rio Grande do Sul, Maranhão, and Ceará. See Anon. 1857a; Anon. 1857b; Anon. 1858a.

22 Act n. 939, 26 September 1857, in article 16, ninth paragraph (Brazil. Ministério do Império 1857 pp. 37–43). In partnership with the Société Zoologique d'Acclimatation, the Brazilian government sought to acclimatize dromedaries to the northeast of the country at the same time the animal regeneration program was being discussed. Incidentally, Burlamaque later contributed to the project by composing a manual to assist the future Brazilian camel farmers. In this manual, which contained a translation of a report composed by the French Society, Buffon was quoted to have determined the “nature of the animal [camelids] does not change, nor does it lend itself to the influences of the climate, &c.” Therefore, it is likely that no contradiction between both projects was perceived (Vogeli 1862, pp. 18–26; Burlamaque 1857, p. 77).

23 Decree n. 2503, 16 November 1859 (Brazil. Ministério do Império 1859a, p. 571).

24 Due to insufficient information about their destination, the values contributed by the provincial governments were left out of the tally.

25 In 1857, the president of the commission was Burlamaque, joined by Manuel Pacheco da Silva, José Bonifácio Nascentes de Azambuja, Joaquim Antônio de Azevedo, Antonio José Gonçalves Fontes, João Carlos de Souza Ferreira, and Augusto Dias Carneiro (Sociedade Auxiliadora... 1857).

26 Burlamaque was a prolific author and published many books, most of them agricultural manuals, see Moreira (1866a, b, pp. 17–18).

27 In 1870 their efforts were turned to a project in direct opposition to Buffon’s degeneration. In a campaign against Chinese immigration, it was argued that “to obtain a happy progeny consists in avoiding the alliances of extremes and in bringing more or less similar races together, and instead of following Buffon's ideas, act on the contrary within more restricted geographical limits” (Moreira 1870, pp. 376–377).

28 Originally Charles Émile Adet, he was born in France, but immigrated to Brazil with his family at a young age. He was employed as a professor of geography, history, French, and Greek, and as a journalist. In the fifties, he became famous when he took over the direction of *Jornal do Commercio*, a major and one of the longest-running newspapers in Brazil (Blake 1895, v. 2, pp. 61–62). Adet was also a member of the Sociedade Auxiliadora and, on August 1st, 1859, sent a copy of his book to its board of directors (Sociedade Auxiliadora… 1859)

29 Adet’s campaign against Burlamaque’s extended to the newspapers, see Anon. (1858b, c, d).

30 Adet transcribed an excerpt from the *Maison rustique du XIX siècle*, albeit without proper reference, where he condemned the idea that linebreeding would necessarily cause degeneration. The excerpt ‘quoted’ is the same excerpt quoted in the first section of this article (Bixio 1837, p. 376; Adet 1858, pp. 67–73).

31 Vogeli graduated from the École nationale vétérinaire d’Alfort, later being hired by the Brazilian government. After spending almost fourteen years in the country, from 1856 to 1870, he returned to France (Neumann 1896, pp. 412–413).

32 Formerly known as Escola Militar and Academia Militar, it was an institution of higher education in Rio de Janeiro (Brazil 1859).

33 Since before its independence, Brazil had a state-funded horse stud known as Coudelaria da Cachoeira do Campo, in Minas Gerais.

34 E.g., Imperial Escola de Medicina Veterinária e de Agricultura Prática (Imperial School of Medicine Veterinary and Practical Agriculture), 1883; Imperial Estação Agronômica de Campinas (Imperial Agronomic Station of Campinas), 1887 (Brazil. Ministério da Agricultura 1865, pp. 10–13; reference the same reports for 1865–1910).

35 For information regarding the poor condition of horses in 19th-century Brazil, see Goulart (1964, pp. 137–154).

36 Small animal sizes and poor health were also observed regarding stockbreeding in North America in the 18th century due to poor nutrition and handling (Bidwell and Falconer 1925, p. 107).

## References

[CR1] Abrantes Marquês de (1857). Circular às assembléas legislativas provinciaes. O Auxiliador Da Indústria Nacional.

[CR2] Abreu e Souza Luiz Jacome de (1875). O Cavallo Criação, educação e hygiene do cavallo militar do Brazil.

[CR3] Absell Christopher David, Tena-Junguito Antonio, Kuntz-Ficker Sandra (2017). The Brazilian export economy, 1822–1913. The first export era revisited: Reassessing its contribution to Latin American economies.

[CR4] Adet Carlos Emílio (1858). Zootecnia aplicada. Hippologia. O cavalo. Raça. Produção. Criação. Hygiene. Exterior.

[CR5] Anon (1812). Relatorio anual feito por Lord Sheffield aos criadores de ovelhas na feira das laãs em Lewes, aos 27 de julho, 1812. Correio Braziliense Ou Armazem Literario.

[CR6] Anon. 1828. Rio de Janeiro 13 de fevereiro de 1828. *Jornal do Commercio*, 14 February.

[CR7] Anon (1835). Observações geraes sobre a aquisição e escolha das raças d’animaes domésticos, etc. O Auxiliador Da Indústria Nacional.

[CR8] Anon (1836). Idêas sobre a criação dos carneiros, modo de os tratar e cuidar. O Auxiliador Da Indústria Nacional.

[CR9] Anon. 1857a. Ensaio sobre a regeneração das raças cavallares do Imperio do Brasil, pelo Dr. F. L. C. Burlamaque. *Dezenove de Dezembro*, 30 September.

[CR10] Anon. 1857b. Regeneração das raças cavallares. *Correio Official de Minas*, 19 October.

[CR11] Anon. 1858a. Ensaio sobre a regeneração das raças cavallares do Brasil pelo Dr. F. L. C. Burlamaque. *O Cearense*, 16 April.

[CR12] Anon. 1858b. O Cavallo. *Jornal do Commercio*, 5 March.

[CR13] Anon. 1858c. A raça cavallar. Resposta ao Sr. G. C. Bellegarde. *Jornal do Commercio*, 2 September.

[CR14] Anon. 1858d. A raça cavallar. Resposta ao Sr. G. C. Bellegarde. *Jornal do Commercio*, 3 September.

[CR15] Azevedo Joaquim Antônio de (1860). Manual do tratamento dos porcos.

[CR16] Azevedo Joaquim Antônio de, Conceição Francisco Corrêa da (1863). Parecer da secção de melhoramento das raças animaes. O Auxiliador Da Indústria Nacional.

[CR17] Bensaude-Vincent Bernadette (2009). A Historical perspective on science and its ‘others’. Isis.

[CR18] Bidwell Percy, Falconer John (1925). History of agriculture in the northern United States, 1620–1860.

[CR19] Bixio Jacques Alexandre (1837). Maison rustique du XIX siècle. Encyclopédie d'agriculture pratique,.

[CR20] Blake Augusto Victorino Alves Sacramento (1895). Diccionario bibliographico brasileiro.

[CR21] Borges Dain (1993). ‘Puffy, ugly, slothful and inert:’ Degeneration in Brazilian social thought, 1880–1940. Journal of Latin American Studies.

[CR22] Brazil (1857). Collecção das leis do Imperio do Brasil de 1857.

[CR23] Brazil (1859). Collecção das leis do Imperio do Brasil de 1859.

[CR24] Brazil (1860). Balanço da receita e despeza do Imperio no exercício de 1857–1858.

[CR25] Brazil (1861). Balanço da receita e despeza do Imperio no exercício de 1858–1859.

[CR26] Brazil. Câmara dos Deputados. 1857. *Annaes do parlamento Brazileiro*. *Câmara dos Srs. Deputados. Primeiro anno da decima legislatura*. *Sessão de 1857*, Vol. III. Rio de Janeiro: Typographia de J. Villeneuve e Com.

[CR27] Brazil. IBGE (1990). Estatísticas históricas do Brasil, séries econômicas, demográficas e sociais, 1550–1985..

[CR28] Brazil. Ministério da Agricultura. 1862. *Ministro (Manoel Felizardo de Souza e Mello) relatório do anno de 1861 apresentado à Assembléa Geral Legislativa...* Rio de Janeiro: Typographia Universal de Laemmert.

[CR29] Brazil. Ministério da Agricultura (1865). Relatorio apresentado à Assembléa Geral Legislativa pelo Ministro (Jesuino Marcondes de Oliveira e Sá) no anno de 1864.

[CR30] Brazil. Ministério da Guerra. 1857. Ministerio da Guerra. Expediente do dia 27 de dezembro de 1856. *Jornal do Commercio*, 1 January.

[CR31] Brazil. Ministério da Guerra. 1874. *Relatorio apresentado à Assembléa Geral Legislativa... pelo Ministro (João José de Oliveira Junqueira) no anno de 1873*. Rio de Janeiro: Imprensa Nacional.

[CR32] Brazil. Ministério da Guerra. 1886. *Relatorio apresentado à Assembléa Geral Legislativa... pelo Ministro (João José de Oliveira Junqueira) no anno de 1885*. Rio de Janeiro: Imprensa Nacional.

[CR33] Brazil. Ministério do Império. 1857. *Relatorio (do anno de 1856) apresentado à Assembléa Geral Legislativa...pelo ministro e secretario d’Estado dos Negocios do Império Luiz Pedreira do Couto Ferraz*. Rio de Janeiro: Typographia Universal de Laemmert.

[CR34] Brazil. Ministério do Império. 1858. *Ministro (Pedro de Araujo Lima) relatório do anno de 1857 apresentado á Assembléa Geral Legislativa...* Rio de Janeiro: Typographia Universal Laemmert.

[CR35] Brazil. Ministério do Império. 1859a. Expediente do dia 15 de outubro de 1859. *Boletim do Expediente do Governo*, October.

[CR36] Brazil. Ministério do Império (1859). Ministro (Sergio Teixeira de Macedo) relatório do anno de 1858 apresentado á Assembléa Geral Legislativa na 3ª sessão da 10ª legislatura.

[CR37] Brazil. Ministério do Império. 1861. Ministerio do Imperio. Expediente do dia 16 de agosto de 1861. *Jornal do Commercio*, 26 August.

[CR38] Britto Fatima (2002). Ciência e público: Caminhos da divulgação científica no Brasil.

[CR39] Buffon, Georges-Louis Leclerc, comte de. 1753. *Histoire naturelle, générale et particuliere, avec la description du cabinet du Roi*, vol. 4. Paris: De l'Imprimerie Royale.

[CR40] Buffon, Georges-Louis Leclerc, comte de. 1761. *Histoire naturelle, générale et particuliere, avec la description du cabinet du Roi*, vol. 9. Paris: De l'Imprimerie Royale.

[CR41] Buffon, Georges-Louis Leclerc, comte de. 1766. *Histoire naturelle, générale et particuliere, avec la description du cabinet du Roi*, vol. 14. Paris: De l'Imprimerie Royale.

[CR42] Buffon, Georges-Louis Leclerc, comte de. 1792. *Barr’s Buffon. Buffon’s natural history: Containing a theory of the Earth, a general history of man, of the brute creation, and of vegetables, minerals, &c. From the French. With notes by the translator*, trans. James Smith Barr. London: J. S. Barr.

[CR43] Burlamaque Frederico Leopoldo César (1856). Ensaio sobre a regeneração das raças cavallares do Imperio do Brasil.

[CR430] Burlamaque, Frederico Leopoldo César. 1856b. *Ensaio sobre a regeneração das raças cavallares do Imperio do Brasil*. 2nd ed. Rio de Janeiro: Typographia Nicolau Lobo Vianna & Filhos.

[CR44] Burlamaque Frederico Leopoldo César (1857). Aclimatacção de dromedário nos sertões do norte do Brazil e da cultura da tamareira, com traducção do rellatorio de Mr. Dareste, apresentada à Sociedade Zoológica de Aclimatação de Paris, sobre o mesmo assumpto.

[CR45] Caponi Gustavo (2009). La miseria de la degeneración: el materialismo de Buffon y las ‘limitaciones’ de su transformismo. Hist. Cienc. Saude-Manguinhos.

[CR46] Cartron Laure, Müller-Wille Staffan, Rheinberger Hans-Joerg (2007). Degeneration and ‘alienism’ in early 19th-century France. Heredity produced: At the crossroads of biology politics and culture 1500–1870.

[CR47] Ceará (1858). Relatório que à Assembléa Legislativa Provincial do Ceará apresentou no dia da abertura da sessão ordinaria de 1858, o excellentissimo senhor Dr. João Silveira de Souza, presidente da mesma província.

[CR48] Chamberlin J. Edward, Gilman Sander L (1985). Degeneration: The dark side of progress.

[CR49] Cobb Matthew (2006). Heredity before genetics: a history. Nature Reviews Genetics.

[CR50] Cribelli Teresa (2016). Industrial forests and mechanical marvels: Modernization in 19th-century Brazil.

[CR51] Davidson Jenny (2009). Breeding: A partial history of the 18th century.

[CR52] Dean Warren (1971). Latifundia and land policy in 19th-century Brazil. Hispanic American Historical Review.

[CR53] Derry Margaret E (2015). Masterminding nature: The breeding of animals, 1750–2010.

[CR54] Domingues, Heloisa Maria Bertol. 1995. *Ciência: um caso de política. As relações entre as ciências naturais e a agricultura no Brasil Império*. PhD thesis, University of São Paulo.

[CR55] Dugatkin Lee Alan (2009). Mr. Jefferson and the giant moose: Natural history in early America.

[CR56] Eddy John H (1994). Buffon's *Histoire naturelle*: History? A critique of recent interprétations. Isis.

[CR57] Eisenberg Peter (1974). The sugar industry in Pernambuco, modernization without change, 1840–1910.

[CR58] Fausto Manoel de Oliveira (1859). Melhoramento das raças de animaes. O Auxiliador Da Indústria Nacional.

[CR59] Félix Regina R, Juall Scott D (2016). Cultural exchanges between Brazil and France.

[CR60] Fernandes Antonio Carlos Sequeira (2010). Uma lembrança de infância: os 'fósseis colossais' e o papel de Frederico Leopoldo César Burlamaque como paleontólogo brasileiro. Filosofia e História Da Biologia.

[CR61] Ferreira Breno Leal (2022). ‘Economia da natureza’, ‘economia animal’ e ‘economia vegetal’ na escrita de naturalistas luso-americanos (1786–1815). Bol. Mus. Pará. Emilio Goeldi. Science Hum..

[CR62] Figueiredo Francisco José de, Absolon Bruno Araujo, Gallo Valéria (2017). Emilio Joaquim da Silva Maia (1808–1859) e o seu ensaio sobre 'Geographia Zoológica'. Filosofia e História Da Biologia.

[CR63] Galfione María Verónica (2013). Natural history and temporalization: Reflections on Buffon’s Natural history. Hist. Cienc. Saude-Manguinhos.

[CR64] Gerbi, Antonello. 2010 [1955]. *The dispute of the New World. The history of a polemic, 1750-1900*, trans. into English by Jeremy Moyle. Pittsburgh: University of Pittsburgh Press.

[CR65] Germiniani Clotilde de Lourdes B (1998). A história da medicina veterinária no Brasil. Archives of Veterinary Science.

[CR66] Glacken Clarence (1967). Traces on the Rhodian shore: Nature and culture in Western thought from ancient times to the end of the eighteenth century.

[CR67] Goulart José Alípio (1964). O cavalo na formação do Brasil.

[CR68] Guest Kristen, Mattfeld Monica (2019). Equestrian cultures: Horses, human Society, and the discourse of modernity.

[CR69] Gutiérrez-Maya Jazmín I, Collazo-Reyes Francisco, Vega y Ortega Rodrigo Antonio (2021). The expansion of modern science through the Catalog of Scientific Papers, XIX century: the Latin American presence. Scientometrics.

[CR70] Hamburger, Amélia Império, ed. et al. 1996. *A ciência nas relações Brasil-França (1850-1950)*. São Paulo: Edusp, FAPESP.

[CR71] Heymann Matthias (2010). The evolution of climate ideas and knowledge. Wires Climate Change.

[CR72] Huxley Robert (2007). The great naturalists.

[CR73] Jones Peter (2016). Agricultural enlightenment: Knowledge, technology, and nature, 1750–1840.

[CR74] Kury Lorelai (2004). Homens de ciência no Brasil: Impérios coloniais e circulação de informações (1780–1810). Hist. Cienc. Saude-Manguinhos.

[CR76] Lacerda João Baptista de (1905). Fastos do Museu Nacional do Rio de Janeiro: Recordações historicas e scientificas fundadas em documentos authenticos e informações verídicas.

[CR77] Lawrence Christopher (2010). Degeneration. The Lancet.

[CR78] Lehleiter Christine (2014). Romanticism, origins, and the history of heredity.

[CR79] Lopes Maria Margaret (1997). O Brasil descobre a pesquisa científica: Os museus e as ciências naturais no século XIX.

[CR80] Lourenço Fernando Antonio (2001). Agricultura ilustrada: Liberalismo e escravismo nas origens da questão agrária Brasileira.

[CR81] Lush Jay Laurence (1943). Animal breeding plans.

[CR82] Machado Gabriel Militão de Vila Nova (1859). Relatório de 1858. O Auxiliador Da Indústria Nacional.

[CR83] Maranhão. 1858. *Relatorio do Exm. Snr. presidente doutor Francisco Xavier Paes Barreto apresentado ao Exm. Sr. vice presidente doutor João Pedro Dias Vieira: Ao passar-lhe a administração no dia 13 de abril de 1858*. Maranhão: Typ. da Temperança.

[CR84] Marques Adílio Jorge, Filgueiras Carlos Alberto Lombardi (2010). A química atmosférica no Brasil de 1790 a 1853. Química Nova.

[CR85] Massarani Luisa, Moreira Ildeu de Castro (2016). Science communication in Brazil: A historical review and considerations about the current situation. Ann. Acad. Bras. Cienc..

[CR86] Matz Brendan (2011). Crossing, grading, and keeping pure: Animal breeding and exchange around 1860. Endeavour.

[CR75] Moll L (1838). Economia do gado. O Auxiliador Da Indústria Nacional.

[CR87] Moreira Nicolau Joaquim (1866). Zootechnia. O Auxiliador Da Indústria Nacional.

[CR88] Moreira, Nicolau Joaquim. 1866b. Elogio histórico pronunciado perante S.M.I. em sessão d´assembléia geral da Sociedade Auxiliadora da Indústria Nacional por ocasião do ato solemne de inauguração do busto do conselheiro Frederico Cezar Leopoldo Burlamaqui. Rio de Janeiro: Typographia da Indústria Nacional de Cotrim & Campos.

[CR89] Moreira Nicolau Joaquim (1870). Discurso pronunciado na sessão da Sociedade Auxiliadora da Industria Nacional, em 16 de Agosto. O Auxiliador Da Indústria Nacional.

[CR90] Moya José C, Cañizares-Esguerra Jorge, Seeman Erik R (2018). Modernization, modernity, and the trans/formation of the Atlantic world in the 19th century. The Atlantic in global history.

[CR91] Müller-Wille Staffan, Müller-Wille Staffan, Rheinberger Hans-Jörg (2007). Figures of inheritance, 1650–1850. Heredity produced: At the crossroads of biology politics and culture 1500–1870.

[CR92] Nelson, William Max. 2021. Generating time: Buffon and the biological instruments of futurity. In *The time of enlightenment: Constructing the future in France, 1750 to year one*, 95–120. Toronto: University of Toronto Press.

[CR93] Neumann Louis Georges (1896). Biographies vétérinaires.

[CR94] Olby Robert, Olby R, Cantor GN, Christie JRR, Hodge MJS (1996). The emergence of genetics. Companion to the history of modern science.

[CR95] Orel Vítězslav, Wood Roger J (1981). Early developments in artificial selection as a background to Mendel's research. History and Philosophy of the Life Sciences.

[CR96] Osborne Michael A (2001). Acclimatizing the world: A history of the paradigmatic colonial science. Osiris.

[CR97] Pallares-Burke Maria Lucia Garcia (1998). A imprensa periódica como uma empresa educativa no século XIX. Cadernos De Pesquisa.

[CR98] Pemberton Neil, Strange Julie-Marie, Worboys Michael, Kean Hilda, Howell Philip (2018). Breeding and breed. The Routledge companion to animal-human history.

[CR99] Penteado David Francisco de Moura (2022). Sociedade Auxiliadora da Indústria Nacional: A ambiguidade de uma associação civil a serviço do Estado brasileiro. Revista Brasileira De História Da Ciência.

[CR100] Perrone-Moisés Leyla (2013). Cinco séculos de presença f rancesa no Brasil: Invasões, missões, irrupções.

[CR101] Rio Grande do Sul (1860). Relatorio apresentado à Assembléa Provincial de S. Pedro do Rio Grande do Sul, na 1.ª sessão da 9.ª legislatura pelo conselheiro Joaquim Antão Fernandes Leão.

[CR102] Roche Daniel (2008). Equestrian culture in France from the 16th to the 19^th^ century. Past & Present.

[CR103] Roger Jacques (1997). Buffon: A life in natural history.

[CR104] Russell Nicholas (1986). Like engend'ing like: Heredity and animal breeding in early modern England.

[CR105] Silva Antonio de Moraes e (1823). Diccionario da língua portugueza.

[CR106] Silva Antonio de Moraes (1890). Diccionario de língua portugueza.

[CR107] Silva César Agenor Fernandes da, Penteado David Francisco de Moura (2017). O perfil dos redatores do periódico O Auxiliador da Indústria Nacional (1833–1896). Revista Diálogos Mediterrânicos.

[CR108] Silva Ignacio Accioli de Cerqueira e (1849). Dissertação historica, ethnographica e politica sobre as tribus aborigenes que habitavam a provincia da Bahia ao tempo em que o Brazil foi conquistado. Revista Trimensal De Historia e Geographia.

[CR109] Silva Innocencio Francisco da (1870). Diccionario bibliographico portuguez.

[CR110] Silva Márcio Antônio Both da (2020). Mudar para permanecer: O atraso da agricultura brasileira sob perspectiva comparada (séculos XIX e XX). Revista De História Comparada.

[CR111] Siqueira Joaquim P. F de (1812). Sobre a creação, e vantagens de gado Cabrum em Portugal. Memorias Economicas Da Academia Das Sciencias De Lisboa.

[CR112] Sloan Philip Reid (1974). The idea of racial degeneracy in Buffon's *Histoire naturelle*. Studies in Eighteenth-Century Culture.

[CR113] Sociedade Auxiliadora da Indústria Nacional (1852). Sessão do dia de 4 de maio de 1852. O Auxiliador Da Indústria Nacional.

[CR114] Sociedade Auxiliadora da Indústria Nacional (1857). Estatuto da Sociedade Auxiliadora da Indústria Nacional.

[CR115] Sociedade Auxiliadora da Indústria Nacional (1859). Sessão do conselho em 1º de agosto de 1859. O Auxiliador Da Indústria Nacional.

[CR116] Sociedade Auxiliadora da Indústria Nacional (1867). Sessão do conselho em 2 de janeiro de 1867. O Auxiliador Da Indústria Nacional.

[CR117] Sociedade Auxiliadora da Indústria Nacional (1869). Estatutos da Sociedade Auxiliadora da Indústria Nacional.

[CR118] Sociedade Auxiliadora da Indústria Nacional (1870). Sessão do conselho em 4 de maio de 1870. O Auxiliador Da Indústria Nacional.

[CR119] Soppelsa Peter, Boddice Rob (2011). The instrumentalisation of horses in 19th-century Paris. Anthropocentrism: Human, animals, environment.

[CR120] Strabo, ed. Jones, Horace Leonard. 1924. *The geography of Strabo*. Cambridge: Harvard University Press.

[CR121] Stein Stanley J (1985). Vassouras, a Brazilian coffee county, 1850–1900.

[CR122] Sweet Timothy, Boyden Michael (2021). The degeneration thesis. Climate and American literature.

[CR123] Taunay Carlos Augusto (1839). Manual do agricultor brazileiro.

[CR124] Thompson Francis M. L (1976). 19th-century horse sense. The Economic History Review.

[CR125] Topham Jonathan R, Papanelopoulou Faidra, Nieto-Galan Agustí, Perdiguero Enrique (2009). Rethinking the history of science popularization/popular science. Popularizing science and technology in the European periphery, 1800–2000.

[CR126] Travieso Emilliano (2022). Soils, scale, or elites? Biological innovation in Uruguayan cattle farming, 1880–1913. Economic History Review.

[CR127] Veloso José Mariano da Conceição (1806). O fazendeiro do Brazil.

[CR128] Vergara Moema de Rezende (2008). Ensaio sobre o termo ‘vulgarização científica’ no Brasil do século XIX. Revista Brasileira De História Da Ciência.

[CR129] Vogeli Felix (1862). Rapport sur le transport à bord du trois-mâts Le Splendide de quatorze chameaux, d'Alger à Fortaleza do Ceara (Brésil). Bulletin De La Société Impériale Zoologique D'acclimatation.

[CR130] Weil Kari (2020). Precarious partners: Horses and their humans in 19th-century France.

[CR131] Wood Roger J, Orel Vítězslav (2005). Scientific breeding in central Europe during the early 19th century: Background to Mendel’s later work. Journal of the History of Biology.

[CR132] Wood Roger J, Müller-Wille Staffan, Rheinberger Hans-Jörg (2007). The sheep breeders’ view of heredity. Heredity produced: At the crossroads of biology politics and culture 1500–1870.

